# Design and Application of Superhydrophobic Magnetic Nanomaterials for Efficient Oil–Water Separation: A Critical Review

**DOI:** 10.3390/molecules30153313

**Published:** 2025-08-07

**Authors:** Rabiga M. Kudaibergenova, Elvira A. Baibazarova, Didara T. Balpanova, Gulnar K. Sugurbekova, Aizhan M. Serikbayeva, Marzhan S. Kalmakhanova, Nazgul S. Murzakasymova, Arman A. Kabdushev, Seitzhan A. Orynbayev

**Affiliations:** 1 Department of Chemistry and Chemical Technology, Faculty of Technology, M. Kh. Dulaty Taraz University, Taraz 080000, Kazakhstan; ea.baibazarova@dulaty.kz (E.A.B.); ms.kalmakhanova@dulaty.kz (M.S.K.); aa.kabdushev@dulaty.kz (A.A.K.); sa.orynbayev@dulaty.kz (S.A.O.); 2 Department of Chemistry, School of Pharmacy, Kazakh National Medical University Named After S.D. Asfendiyarov, Almaty 050000, Kazakhstan; balpanova.d@kaznmu.kz; 3 Department of Chemistry, Faculty of Natural Sciences, Eurasian National University Named After L.N. Gumilyov, Astana 010000, Kazakhstan; gulnar.sugurbekova@nu.edu.kz; 4 Department of Chemistry and Pharmaceutical Engineering, M. Auezov South Kazakhstan Research University, Shymkent 160012, Kazakhstan; aizhan.serikbaeva@auezov.edu.kz

**Keywords:** superhydrophobic materials, magnetic nanomaterials, oil–water separation, surface engineering, nanostructured sorbents, environmental remediation

## Abstract

Superhydrophobic magnetic nanomaterials (SHMNMs) are emerging as multifunctional platforms for efficient oil–water separation due to their combination of extreme water repellency, strong oil affinity, and external magnetic responsiveness. This review presents a comprehensive analysis of recent advances in the design, synthesis, and environmental application of SHMNMs. The theoretical foundations of superhydrophobicity and the physicochemical behavior of magnetic nanoparticles are first outlined, followed by discussion of their synergistic integration. Key fabrication techniques—such as sol–gel synthesis, electrospinning, dip-coating, laser-assisted processing, and the use of biomass-derived precursors—are critically assessed in terms of their ability to tailor surface morphology, chemical functionality, and long-term durability. The review further explores the mechanisms of oil adsorption, magnetic separation, and material reusability under realistic environmental conditions. Special attention is paid to the scalability, mechanical resilience, and environmental compatibility of SHMNMs in the context of water treatment technologies. Current limitations, including reduced efficiency in harsh media, potential environmental risks, and challenges in material regeneration, are discussed. This work provides a structured overview that could support the rational development of next-generation superhydrophobic materials tailored for sustainable and high-performance separation of oil and organic pollutants from water.

## 1. Introduction

Oil spills and oily wastewater generated from petroleum extraction, refining, and transportation activities pose a serious threat to the aquatic environment and public health. The frequent occurrence of industrial effluents containing hydrocarbons and organic solvents not only deteriorates water quality but also leads to long-term ecological damage [1]. According to the International Tanker Owners Pollution Federation (ITOPF), more than 7000 oil spill incidents have occurred globally since the 1970s, emphasizing the urgent need for advanced oil–water separation technologies [2].

Conventional methods such as gravity separation, centrifugation, flotation, chemical coagulation, and filtration are commonly employed to address this problem. However, these methods are often energy-intensive, inefficient for stable emulsions, and prone to generating secondary pollution [3]. For example, chemical coagulants can lead to excessive sludge formation, while membrane-based filtration systems suffer from fouling and require frequent maintenance [4]. Therefore, there is an increasing demand for materials and systems that are selective, efficient, reusable, and environmentally friendly.

In this context, materials with superhydrophobic and superoleophilic properties have gained substantial attention. These materials repel water while preferentially adsorbing oil, owing to their hierarchical roughness and low surface energy, inspired by natural surfaces such as lotus leaves and water strider legs [5]. The incorporation of magnetic components further enhances their functionality, enabling remote recovery and reuse through external magnetic fields [6].

Recent advancements in carbon-based nanomaterials such as carbon nanotubes (CNTs), graphene oxide (GO), and reduced graphene oxide (rGO) have facilitated the development of high-performance superhydrophobic magnetic nanocomposites. These materials offer high surface area, chemical durability, ease of surface modification, and compatibility with magnetic nanoparticles like Fe_3_O_4_ [7]. For instance, Zhang et al. fabricated a reduced graphene oxide-based superhydrophobic magnetic sponge (rGO@Fe_3_O_4_) which demonstrated excellent oil absorption capacity and recyclability for up to 10 cycles with minimal loss of efficiency [8]. In another study, Song et al. developed CNT-based magnetic nanocomposites with polydimethylsiloxane (PDMS) that exhibited oil adsorption capacities of over 40 g/g and showed robust stability under harsh pH and temperature conditions [9].

Comparatively, polymer-based sponges and foams (e.g., polyurethane and melamine sponges) coated with hydrophobic agents like silanes or fatty acids are widely used due to their low cost and scalability. However, these materials often suffer from mechanical degradation, poor chemical resistance, and limited reusability [10]. In contrast, carbon-based and metal oxide composites show significantly improved mechanical strength, magnetic responsiveness, and environmental durability, making them better suited for industrial applications [11].

Magnetic separation not only simplifies the recovery process but also minimizes energy consumption and operational complexity. Hojatjalali et al. reported a magnetic melamine sponge modified with ZnO, stearic acid, and Fe_3_O_4_ nanoparticles that achieved a water contact angle of 160.96° and oil adsorption capacities up to 83.71 g/g. Moreover, it maintained its efficiency over 10 reuse cycles in real industrial wastewater [12].

Despite these promising results, several challenges remain, including the scalability of synthesis methods, biocompatibility of materials, and long-term stability in real-world conditions. Environmental concerns regarding the potential leaching of nanomaterials and the toxicity of surface modification agents must also be addressed. There is a growing interest in developing green fabrication strategies using biobased polymers, non-toxic reagents, and low-energy processes [13].

This review aims to critically assess the recent progress in the design, synthesis, and application of superhydrophobic magnetic nanomaterials for oil–water separation. We will focus on the material composition, fabrication strategies, physicochemical properties, and practical application performance, while identifying key limitations and future research directions to bridge the gap between laboratory-scale innovation and real-world deployment.

In preparing this critical review, we systematically selected and analyzed peer-reviewed journal articles and authoritative book chapters, focusing primarily on works published from 2015 onward. Studies were included if they reported on superhydrophobic magnetic nanomaterials (SHMNMs) applied to oil–water separation, with preference given to those evaluating performance in real or simulated environmental conditions. Articles highlighting material limitations, environmental risks, and scalability were prioritized to provide a balanced and critical perspective. Reviews and reports lacking original data, or focused solely on non-magnetic or non-superhydrophobic materials, were excluded. This approach allowed us to synthesize current advances while critically assessing unresolved challenges and future directions.

## 2. Theoretical Foundations of Superhydrophobic Magnetic Nanomaterials

### 2.1. Definition and Principles of Superhydrophobicity

Superhydrophobic surfaces are characterized by minimal wetting, where water droplets form nearly perfect spheres and roll off easily even at slight inclines, providing a self-cleaning effect. This behavior results from the combination of micro- and nanoscale surface structures with hydrophobic chemical modification.

The phenomenon of superhydrophobicity is widespread in nature, the most striking example of which are lotus leaves (*Nelumbo nucifera*), which have the ability to remain clean due to the complex micro- and nanostructure of their surface in combination with low surface energy [14]. The study of natural systems formed the basis for the creation of artificial superhydrophobic materials.

The mechanisms of superhydrophobic behavior are described by two main models: Wenzel and Cassie–Baxter. According to the Wenzel model, an increase in the microroughness of the surface leads to an increase in its initial hydrophobicity due to an increase in the effective contact area between the solid and the liquid [15]. Meanwhile, the Cassie–Baxter model considers the case when the liquid does not interact directly with the surface of the material, but with a composite surface consisting of a solid substance and trapped air pockets. This ensures extremely low adhesion of water to the surface and facilitates easy rolling of droplets.

To implement a superhydrophobic state, two conditions must be met: the presence of a multilevel micro- and nanostructure that forms the necessary roughness, and surface modification with compounds with low surface energy. Fluorinated alkoxysilanes, long-chain alkyls or fatty acids, which ensure minimal interaction of water molecules with a solid surface [16], are often used as such modifiers.

In the context of oil and water separation tasks, superhydrophobic materials are additionally characterized by superoleophilicity, the ability to effectively wet oil. This combination of properties allows for the selective extraction of oil contaminants from aqueous systems, ensuring high cleaning efficiency even in the case of stable emulsions and microdroplet dispersions.

Thus, superhydrophobicity is the result of an optimal combination of surface topography and chemical composition, providing unique performance characteristics that are in demand in environmental, energy, and biomedical technologies.

### 2.2. Physicochemical Properties of Magnetic Nanomaterials

Magnetic nanomaterials (MNM), especially those based on iron oxides, are a unique class of functional compounds with a number of specific physicochemical properties: high specific surface area, magnetic responsiveness, controlled morphology, and the possibility of precise surface modification. These properties determine their wide application in various technological and environmental processes, including biomedicine, catalytic reactions, targeted delivery of substances and, in particular, in superhydrophobic magnetic nanocomposites for efficient separation of oil and water.

The key property of MNM is their magnetic behavior, which differs significantly from bulk materials. The most widely used nanoparticles are based on magnetite (Fe_3_O_4_) and maghemite (γ-Fe_2_O_3_). When the particle size decreases to 10–20 nm, they enter a state of superparamagnetism, a state in which the particles exhibit high magnetic susceptibility in an external field, but do not retain magnetization after its removal [17,18]. This ensures stable colloidal dispersion without aggregation and allows the material to be reused without deterioration of the sorption properties.

Superparamagnetic particles are especially valuable in sorption systems, since they can be easily and quickly extracted from an aqueous medium with an external magnet, without the need for centrifugation or filtration. In addition, the absence of residual magnetization prevents the formation of aggregates, which is important for repeated use of the sorbent [19].

The shape and size of MNMs have a direct impact on their magnetic and adsorption properties. With decreasing particle diameter, the surface-to-volume ratio increases, which contributes to an increase in the number of available active sites and enhances interaction with pollutants. However, excessive reduction in size can lead to instability and loss of magnetic sensitivity [20]. For sorption applications, spherical or quasi-spherical particles with a diameter of 10–30 nm are usually used, providing a compromise between high activity and stability. It is also important to note that controlled morphology (e.g., cubic, octahedral, flower structures) allows for control of crystallographic orientation, which in turn affects magnetic anisotropy and catalytic properties [21].

The chemical stability of NMNs determines their suitability for use in real aqueous and wastewater environments. Magnetite (Fe_3_O_4_) is stable in neutral and slightly acidic solutions, but can be oxidized to γ-Fe_2_O_3_ under the influence of oxygen, which is accompanied by a decrease in magnetization [22]. To prevent such processes, surface passivation methods are used: coating particles with silicone, carbon, polymers (e.g., PEG, PDMS), or organic ligand shells [23]. The coatings not only protect against oxidation, but also allow the creation of hydrophobic surfaces, which is especially important in systems for removing organic compounds from water. For example, modification of Fe_3_O_4_ using stearic acid, fluorosilanes, or graphene-like materials significantly improves the hydrophobicity and stability of the nanocomposite [24].

Surface functionalization is one of the main advantages of MNMs. Due to the presence of hydroxyl groups on the surface of iron oxides, their covalent modification with a wide range of compounds is possible: from simple fatty acids to complex polymers and biomolecules. Functionalization can be aimed at both improving compatibility with the matrix (for example, a polymer sponge or carbon network) and ensuring selective interaction with target pollutants [25].

In the context of superhydrophobic systems, surface modification plays a crucial role: it is what ensures water repellency, resistance to pollution, and reuse. For example, Lv et al. (2019) proposed a method for producing rGO/Fe_3_O_4_-based aerogels with a modified hydrophobic shell, demonstrating high efficiency of selective absorption of organic solvents [26].

One of the main problems associated with the use of MNMs in aqueous media is their tendency to agglomerate. Aggregation reduces the active surface, worsens distribution in solution, and reduces the efficiency of interaction with pollutants. To prevent agglomeration, electrostatic stabilization (for example, due to surface charge) and steric stabilization (using polymer shells) are used [27].

The development of multifunctional MNMs with stable dispersion properties and controlled magnetic reactions remains one of the priority tasks in the field of creating new-generation intelligent sorbents.

Thus, the physicochemical properties of magnetic nanomaterials determine their efficiency and prospects in the problems of selective separation, including in the field of purification of aqueous media from oil products and organic solvents. Their high specific surface area, superparamagnetic properties, chemical inertness, and wide modification possibilities make such materials an ideal basis for creating superhydrophobic sorbents with magnetic controllability. Future research should focus on ensuring environmental safety, resistance to extreme operating conditions, and scalability of synthesis processes.

### 2.3. Synergistic Combination of Superhydrophobicity and Magnetic Properties

One of the most promising areas in the creation of new-generation functional sorbents is the combination of such properties as superhydrophobicity and magnetic responsiveness in one material. Each of these properties in itself opens up significant opportunities in the field of selective separation of liquids and purification of aqueous media. However, only in their synergistic combination can we implement materials that have not only high sorption capacity for organic pollutants, but also the ability to quickly extract and reuse without significant loss of efficiency.

From a scientific point of view, the synergy between superhydrophobicity and magnetic properties is based on the simultaneous control of two key parameters: the surface energy and magnetic sensitivity of the material. Superhydrophobicity is provided by a hierarchically organized surface (usually micro- and nanostructured) in combination with chemically inert and water-repellent groups, such as fluorine-containing or alkyl radicals. On the other hand, magnetic properties are due to the inclusion of iron oxide nanoparticles in the structure of the material, most often magnetite (Fe_3_O_4_) or maghemite (γ-Fe_2_O_3_), which exhibit superparamagnetic behavior when the size is reduced to critical values of about 10–20 nm [28].

The combination of superhydrophobicity and magnetic responsiveness in a single nanostructure is an example of a pronounced synergistic effect, in which the properties of each component are not only preserved, but also enhanced due to their mutual interaction. From a scientific point of view, we are talking about the creation of multifunctional sorbents with a high degree of selectivity, structural stability, controllability, and reusability potential. Despite the significant amount of experimental data presented in recent years, a number of fundamental issues—such as surface stability during multiple sorption cycles, the interaction between the magnetic and hydrophobic domains, and the scalability of the technologies—remain open and require in-depth analysis.

The concept of synergy suggests that the combined effect exceeds the sum of individual properties. In the context of superhydrophobic magnetic nanomaterials (SHMNM), this is expressed, for example, in the fact that the adsorption capacity with respect to organic compounds is higher than that of individual magnetic or hydrophobic systems, and the process of sorbent regeneration becomes simpler and more efficient due to targeted magnetic extraction. However, the key condition is the balance of characteristics: excessive addition of magnetic nanoparticles can disrupt the hydrophobic morphology of the surface, and an excess of a hydrophobic modifier can reduce the mobility and dispersibility of magnetic components.

Magnetic nanoparticles are usually represented by superparamagnetic Fe_3_O_4_, which is stable in aqueous systems and is easily modified. However, with uncontrolled synthesis and the absence of protective layers (silica, polymers, carbon), such particles are prone to oxidation to γ-Fe_2_O_3_, accompanied by a decrease in magnetization [29]. In addition, Fe_3_O_4_ in an aqueous medium exhibits a tendency to aggregate due to magnetic interaction, which negatively affects the homogeneity of distribution and sorption activity of the material. This requires the mandatory inclusion of stabilizers and modifiers, such as oleic acid, PEG, sodium stearate, or surfactants containing fluorine [19].

Superhydrophobic properties are known to be realized through a combination of two factors: the presence of multiscale surface roughness and low surface energy. Most effective SHMNMs are based on composite structures: polymer sponges, graphene networks, carbon nanotubes, or aerogels. Of particular interest is the approach implemented by Cao et al. (2017) [30], which used reduced graphene oxide (rGO) functionalized with magnetic nanoparticles and stearic acid. The authors showed that the resulting material exhibited high adsorption of organic solvents (up to 90 g/g), was resistant to extreme pH values, and retained superhydrophobicity even after 10 regeneration cycles [30]. However, despite the high productivity, the aerogel structure in this system remains fragile, and its mechanical stability during long-term use is questionable.

It is critically important to note that the nature of the bond between the hydrophobic matrix and the magnetic particles directly affects the stability and efficiency of the sorbent. In the case of physical adsorption of magnetic nanoparticles on the surface of a hydrophobic substrate, their loss during operation is almost inevitable. Chemical immobilization (via silane agents, grafted monomers, or hybrid bridges) allows for increased stability, but may affect magnetic susceptibility due to partial shielding of surfaces [23]. Therefore, the choice of binding method should take into account the nature and purposes of the sorbent application.

The literature also provides examples of integrating magnetic particles directly into the sorbent structure. In the study by Turco et al. (2024) [31], a CNT@Fe_3_O_4_/PDMS material was synthesized, where Fe_3_O_4_ nanoparticles were uniformly distributed in a polydimethylsiloxane matrix modified with carbon nanotubes. This structure provided high mechanical stability, resistance to compression, and resistance to organic solvents. Moreover, the sorbent retained 95% of its adsorption capacity after 20 cycles of use [31]. However, the disadvantage of this system was a decrease in magnetic sensitivity with an increase in the proportion of PDMS, which once again emphasizes the importance of an optimal balance between the components.

This structure provided high mechanical stability, resistance to compression, and resistance to organic solvents. Moreover, the sorbent retained 95% of its adsorption capacity after 20 cycles of use [31]. However, the disadvantage of this system was a decrease in magnetic sensitivity with an increase in the proportion of PDMS, which once again emphasizes the importance of an optimal balance between the components.

**Figure 1:**
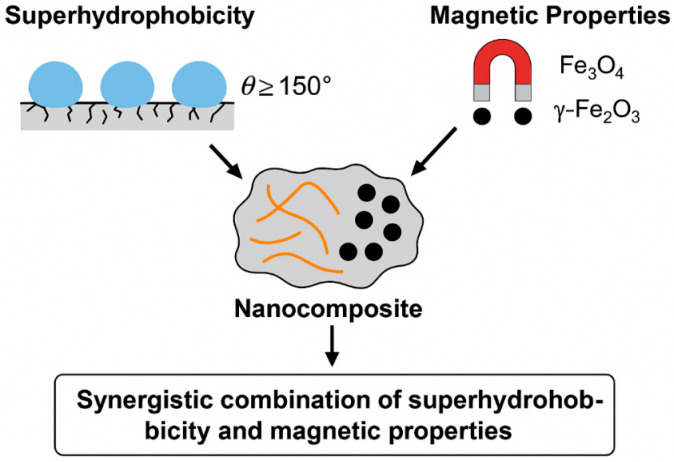
Schematic representation of the structure of a superhydrophobic magnetic nanocomposite designed for oil–water separation.

The generalized scheme shown in Figure 1 allows visualization of the structural organization of such composites, and shows the architecture of a typical superhydrophobic magnetic material. The hydrophobic matrix with hierarchical roughness contains embedded magnetic nanoparticles (most often Fe_3_O_4_), and its surface is additionally modified with low-energy functional groups. This structure provides simultaneous water repulsion, high sorption of organic liquids, and the ability to extract the sorbent under the influence of a magnetic field. This triple functionality demonstrates the real synergy that underlies highly efficient oil–water separation systems.

The diagram illustrates the structure of a superhydrophobic magnetic nanocomposite comprising a porous matrix (e.g., sponge or aerogel) with a hierarchically rough surface. Magnetic iron oxide (Fe_3_O_4_) nanoparticles are incorporated into the matrix, imparting magnetic responsiveness. The surface is further functionalized with hydrophobic groups (such as alkylsilylenes or stearic acid), resulting in water repellency and oil absorption. The material can be removed from the medium by applying an external magnetic field.

A comparative analysis of existing works shows that the most stable and effective SHMNMs have the following general characteristics: a multilayer structure, stable covalent bonds between components, high specific surface area (at least 100 m^2^/g), internal porosity, and a wide range of thermal and chemical stability. However, the issues of scalability of synthesis, reproducibility of properties with increasing volumes, and the economic feasibility of using expensive modifiers and precursors remain unresolved.

Thus, the synergistic combination of superhydrophobic and magnetic properties in a single nanostructure is not only a scientifically sound but also a technologically promising concept for creating reusable sorbents for environmental problems. However, their successful implementation in industry requires further improvement of synthesis methods, improvement of mechanical strength, and development of universal approaches to control the distribution and interaction between magnetic and hydrophobic phases.

## 3. Methods of Synthesis and Modification of SHMNMs

### 3.1. The Basis of Superhydrophobic Materials

Superhydrophobic materials are engineered systems with exceptional water-repellent properties. The basis of such materials is formed by a delicate balance between the micro- and nanotopography of the surface and its chemical composition. Their creation is impossible without taking into account two interrelated factors: the structural architecture of the surface and its energy characteristics. Natural analogs such as lotus leaves, insect wings, and feathers of waterfowl demonstrate that the key condition for superhydrophobicity is the presence of a hierarchical surface texture supplemented by a layer of low-energy functional groups [32].

Achieving stable superhydrophobicity requires a two-level surface morphology: microroughness (1–10 μm) combined with nanostructuring (10–100 nm), which traps air between the solid surface and the water droplet. According to the Cassie–Baxter model, this configuration minimizes the liquid–solid contact area, producing high contact angles (>150°) and low roll-off angles (<10°). In contrast, the Wenzel model assumes complete wetting of the surface relief, which diminishes self-cleaning and contamination resistance, making the Cassie–Baxter state more desirable for practical applications [33].

The materials that form the basis of superhydrophobic surfaces can be divided into several classes: inorganic (metal oxides, silica), organic polymers (e.g., polyurethane, PDMS), and carbon materials (graphene, carbon nanotubes). Each of these classes has its own advantages and limitations, both in terms of stability and the possibility of modification. For example, silica coatings are easily structured, but are often brittle and do not withstand mechanical loads. Polymer bases, on the contrary, are highly flexible, but require additional modification to impart the necessary texture [34].

A special category is made up of carbon nanomaterials, such as graphene and multi-walled carbon nanotubes (MWCNTs). Their high specific surface area, chemical inertness, and surface functionalization ability make them extremely promising components in the creation of superhydrophobic coatings. As shown in the paper [35], a superwater-repellent magnetic porous sponge was obtained based on carbon nanotubes (CNTs), NiFe_2_O_4_ nanoparticles, and polydimethylsiloxane (PDMS). CNTs were synthesized by chemical osmosis with a catalyst of nickel ferrites on aluminum hydroxide. The NiFe_2_O_4_ nanostructure obtained by the sol–gel method was used as magnetic nanoparticles. The sponge was created by immersing porous polyurethane in a composition with CNTs and NiFe_2_O_4_, covering it with a PDMS solution. The results showed that the sponge had high hydrophobicity (contact angle of 155.5°), high efficiency of separation of oils and organic liquids (up to 99.81%), good moisture resistance, and the possibility of repeated use (more than 10 cycles). It quickly (about 10 s) and selectively absorbed oil and organic solvents, making it promising for cleaning water with oil pollution.

The choice of base also determines the adhesion of the coating to the substrate and its durability. In the case of solid inorganic substrates (e.g., metal, glass), preliminary surface treatment is often required—sandblasting, plasma, or chemical—to ensure reliable fixation of the functional layer. In addition, it is necessary to consider the compatibility of the base material with modification methods: for example, heat-sensitive polymers do not withstand the high-temperature treatment required to cure silicone coatings.

The chemical composition of the surface layer is no less important. The most widely used fluorine-based compounds are fluorosilanes, fluoropolymers, and perfluorinated organic molecules with minimal surface energy (<20 mN/m). However, their high cost, environmental toxicity, and limited biodegradability have stimulated the development of alternative hydrophobic agents based on waxes, fatty acids, alkyl siloxanes, and biopolymers (e.g., chitosan) [36].

It is important to note that stable superhydrophobicity cannot be achieved by a hydrophobic coating alone—it requires a stable topography. This is why most modern methods are aimed at creating a hierarchical structure followed by chemical treatment. For example, Chen et al. (2009) [37] used a two-step approach: forming a porous structure by electrospinning, followed by modification with fluorinated organosilanes. The resulting surfaces retained superhydrophobic properties after 30 cycles of mechanical abrasion [37].

Comparative characteristics of bases for superhydrophobic materials are given in Table 1.

Thus, the basis of superhydrophobic materials is determined not only by the chemical composition and morphology of the surface, but also by their interaction with each other. Modern research is aimed at finding combinations that ensure the coating’s resistance to external influences (temperature, mechanical, chemical), maintaining superhydrophobicity during long-term use, and the environmental safety of the components used.

### 3.2. Methods of Synthesis of SHMNMs

The creation of superhydrophobic materials requires a finely coordinated approach that combines the formation of a multi-level micro- and nanostructured surface with the modification of its chemical composition. The methods for synthesizing such coatings are conventionally divided into two categories: physical and chemical. In addition, the choice of matrix, substrate, and hydrophobic agent is of great importance, since the effectiveness of the final material is determined by their combination.

#### 3.2.1. Sol–Gel Method

One of the most widely used approaches is the sol–gel synthesis method, especially in the case of inorganic materials. The sol–gel method remains one of the most versatile and widely used approaches for creating superhydrophobic coatings due to its simplicity, low temperature requirements, and the ability to form hierarchical structures [44].

In a recent study, a superhydrophobic surface was developed based on spherical silica nanostructures modified with organosilanes. Using tetraethoxysilane (TEOS) as a precursor and polydimethylsiloxane (PDMS) for surface modification, a water contact angle of up to 162° was achieved, indicating a high degree of hydrophobicity of the coating. This approach has demonstrated its effectiveness in creating stable coatings on glass substrates [45].

The researchers developed hybrid coatings that combined superhydrophobic and anti-corrosion properties using a sol–gel process. The coatings were synthesized based on TEOS, 3-glycidoxypropyltrimethoxysilane (GPTMS), titanium tetrabutoxide (TBT), and the fluorine-containing monomer PFOTES. The resulting coatings demonstrated high water repellency and corrosion resistance when applied to metal substrates [46].

Other studies have shown that the choice of solvent in the sol–gel process has a significant impact on the properties of the resulting aerogels. Using ethanol as a solvent resulted in superhydrophobic silica aerogels, while using water resulted in super-hydrophilic structures. This opens up the possibility of fine-tuning the properties of the materials depending on the requirements of a particular application [47].

The sol–gel method is also successfully used to create superhydrophobic coatings on wood and textile materials. For example, coatings for wood have been developed that provide not only water-repellent properties, but also resistance to mechanical impacts, which makes them promising for use in construction and furniture [48].

#### 3.2.2. Electrospinning

Electrospinning remains one of the most promising methods for creating superhydrophobic coatings due to its ability to form nanofibrous structures with a high degree of control over morphology. Electrospinning is a technological process for producing ultra-thin fibers (with a diameter from tens of nanometers to several micrometers) from polymer solutions or melts under the influence of a high-voltage electrostatic field. The method is based on drawing a stream of liquid from a capillary or syringe containing a polymer solution under the influence of an electric field between a metal nozzle (needle) and a grounded collector. Under the influence of high voltage, a drop of solution at the exit of the needle takes the form of a so-called Taylor cone. When the critical voltage is reached, a stream breaks out from the top of the cone, which is drawn out in the air, loses solvent, and settles in the form of fibers on the receiving surface [49].

Recent studies have demonstrated the possibility of obtaining superhydrophobic coatings with water contact angles exceeding 160° using electrospinning of polymer solutions. For example, in one study, polycaprolactone (PCL)-based coatings modified with fluorine-containing compounds were obtained that demonstrated resistance to mechanical stress and retained their properties after repeated abrasion cycles [50].

Electrospinning also allows the integration of various functional nanoparticles into the fiber structure, which expands the functionality of the resulting coatings. For example, the introduction of graphene oxide (GO) or magnetic nanoparticles (Fe_3_O_4_) into polymer matrices allows the creation of coatings with additional properties, such as electrical conductivity or magnetic sensitivity, while maintaining superhydrophobic characteristics [51].

Electrospinning is widely used in the creation of materials for the effective separation of oil and water. Coatings obtained using this method demonstrate not only a high degree of hydrophobicity, but also resistance to various environmental conditions, which makes them promising for use in wastewater treatment and oil spill response [52,53,54].

#### 3.2.3. Dip-Coating Method

The dip-coating method remains one of the most effective and simple ways to create superhydrophobic coatings due to its versatility and scalability. Dip-coating is a widely used technology for applying thin films and functional coatings based on controlled immersion of a substrate into a solution containing active components, followed by its extraction and drying. The process includes several key stages: (1) immersion of the substrate at a given rate into the working solution, (2) holding in the solution to form an adsorption layer, (3) extraction at a constant rate, forming a uniform film due to gravitational flow, and (4) evaporation of the solvent and heat treatment if necessary [55].

The process of obtaining the modified chalk sponge involves the use of the dip-coating method. After the original sponge is prepared, it is immersed in a solution containing ZnO, Fe_3_O_4_, and stearic acid nanoparticles, which ensures a uniform coating of the sponge surface. This process takes several hours and creates a surface with superhydrophobic and magnetic properties. After coating, the sponge is dried and supplemented with additional characteristics, such as measuring water absorption and magnetic properties, to confirm successful modification [12].

In another study, a superhydrophobic stainless steel mesh coated with polystyrene and SiO_2_ nanoparticles was developed using an immersion method. The resulting mesh had a water contact angle of 158.5 ± 2° and demonstrated separation efficiency of up to 99.33% for low-viscosity oils and 86.66% for high-viscosity oils. The mesh also retained its properties after mechanical tests including bending, twisting, and abrasion [56].

The researchers also developed durable superhydrophobic coatings using a combination of differently sized SiO_2_ nanoparticles, fluorinated alkyl silanes, and polydimethyl siloxane (PDMS). The resulting coatings exhibited water contact angles greater than 175°; resistance to abrasion, UV radiation, and chemical attack; and retained their properties in a variety of aggressive conditions [57].

#### 3.2.4. Laser–Chemical Processing

Laser–chemical processing (LCP) is a method that combines laser radiation with chemical reactions to modify the surface of materials. In the context of creating superhydrophobic magnetic nanomaterials, LCP is used to form micro-nano-structures on the surface, which leads to a change in wettability and imparts magnetic properties [58].

A study by Athanasios Milionis and colleagues describes the development of magnetic nanocomposite sheets with superhydrophobic and superoleophilic surfaces obtained by laser ablation. Polydimethylsiloxane (PDMS) elastomer films containing 2% carbon-coated iron nanoparticles were irradiated with a 248 nm UV laser. Laser treatment induced chemical and structural changes at the micro- and nanoscale, imparting superhydrophobic properties to the surface. The presence of magnetic nanoparticles improved the absorption of UV radiation, facilitating the ablation process and imparting magnetic properties to the material, allowing it to be manipulated using an external magnetic field [59].

Another study describes the use of ultrafast laser machining to create superhydrophobic and superoleophilic surfaces on metal meshes. Laser ablation forms micro-nano-structures on the surface, increasing its roughness and changing wettability [60]. Such treated meshes demonstrate high efficiency in oil–water separation, providing fast and selective filtration.

In the work of Weng et al. (2024) [61], a universal method for creating superhydrophobic surfaces is presented, combining femtosecond laser processing and chemical modification. The method was developed for obtaining water-repellent coatings independent of the type of substrate (metal, glass, polymer). In the first stage, the surface is processed with a femtosecond laser (800 nm, 150 fs), resulting in the formation of a micro-nano-relief. This provides the roughness necessary to retain air between the surface and a water droplet (Cassie–Baxter state). The samples are immersed in a 1% stearic acid solution in ethanol for 2 h, then dried at 60 °C to reduce surface energy. The combined micro/nanostructuring and chemical modification result in a water contact angle > 150°. The coating remains stable after heating, washing, and storage for up to 60 days. The method’s versatility and durability make it promising for oil–water separation, material protection, and self-cleaning applications [61].

#### 3.2.5. Using Biomass

This technique is based on the use of biomass, a renewable and affordable raw material, to create superhydrophobic and magnetic materials. Biomass, such as agricultural waste, wood materials or plant extracts, is thermally or chemically treated to produce porous structures with a high surface area. These structures are then modified with nanoparticles (e.g., tin, iron, or copper oxide) and hydrophobic agents (e.g., stearic acid) to impart superhydrophobic and magnetic properties. The resulting materials are able to effectively separate oil from water by absorbing oils and repelling water [62,63].

One study describes the creation of a biomass aerogel with superhydrophobic and magnetic properties. In this study, a biomass-based superhydrophobic aerogel with integrated magnetic properties was developed for efficient oil–water separation. Plant biomass carbonized at about 500–600 °C in an inert atmosphere was used as a feedstock. The resulting porous carbon in the form of a spongy structure served as a matrix with a developed specific surface area. To impart magnetic properties, magnetite (Fe_3_O_4_) nanoparticles synthesized in situ by co-precipitation of Fe^2+^ and Fe^3+^ salts in an alkaline medium were introduced into the aerogel structure. The resulting magnetic carbon was chemically modified by treating the surface with a stearic acid solution in ethanol to lower its surface energy. The aerogel exhibited high oil sorption capacity (up to 45 g/g), separation efficiency above 99.8%, and retained its performance over more than 10 reuse cycles. The material was easily removed from the solution using an external magnet, making it suitable for mobile cleaning systems [64].

In the second study, a superhydrophobic filtration membrane based on a fabric substrate treated with biogenic tin oxide nanoparticles (Bio-SnO_2_) was developed. A green extract from sunflower leaves (*Helianthus annuus* L.) containing natural reducing agents and stabilizers (phenols, flavonoids) was used to synthesize the nanoparticles. The extract was added to an aqueous SnCl_2_ solution, and controlled growth of tin oxide nanoparticles occurred upon heating to 90 °C. The resulting nanoparticles were applied to cotton fabric by impregnation and drying. Then, the fabric surface was modified with stearic acid to provide superhydrophobic properties. The membrane demonstrated a water wetting angle of 152° and a sliding angle of <5°, ensuring efficient gravity separation of organic and aqueous phases. The sorption values were as follows: diesel—63.5 g/g, coconut oil—70.4 g/g. The material demonstrated resistance to bending, mechanical and alkaline/acidic effects, retained its properties after 10 cycles, and operated in the pH range from 3 to 11. The filtration flow reached 9400 L m^−2^ h^−1^, which makes such a membrane applicable in industry [65].

In another study, powder obtained from crushed and dried corn cobs was used as a matrix. The biomaterial was heat-treated at 300 °C to remove organic compounds and increase the stability of the structure. Then it was mixed with a solution of FeCl_2_ and FeCl_3_, and alkaline precipitation was carried out to obtain Fe_3_O_4_ nanoparticles directly on the surface of the biomaterial particles. Then the resulting magnetic powder was dried and modified with PDMS (polydimethylsiloxane) in a toluene solution at 70 °C. This ensured the formation of a protective hydrophobic layer. The resulting sorbent had superhydrophobicity and magnetic controllability, absorbed oils up to 42 g/g, and retained its efficiency after eight cycles of operation [66].

Table 2 presents a comparative description of the methods considered.

The comparative analysis shows that each of the considered methods has its own advantages and limitations in the context of obtaining superhydrophobic magnetic nanomaterials for oil and water separation. Sol–gel and laser–chemical processing provide high precision and stability of coatings, but require harsh synthesis conditions [45,46,61]. Electrospinning and the immersion method are distinguished by technological flexibility and scalability, especially when creating membranes and sponges [50,51,56,57]. The use of biomass is an environmentally friendly and cost-effective approach that provides high sorption capacity and magnetic controllability [64,67,68]. The optimal choice of method depends on the requirements for the substrate, availability of raw materials, the level of equipment, and the target area of application.

## 4. Application of SHMNMs in Oil and Water Separation

**Figure 2:**
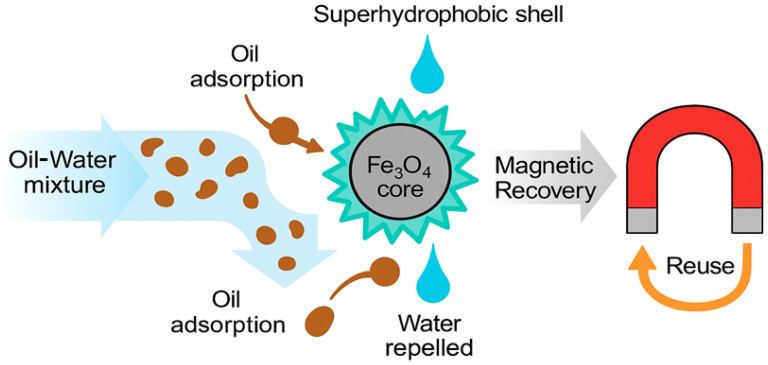
Application of SHMNMs in Oil–Water Separation.

The use of superhydrophobic magnetic nanomaterials in oil–water separation is a promising direction in the field of wastewater treatment and oil spill response (Figure 2).

### 4.1. Sorbents

In the study [69], a new magnetic hydrophobic sorbent based on the ZS@BIF complex was developed and fully characterized. The analytical methods carried out, including XRD, SEM, and EDS, confirmed the correct structure and uniform distribution of zinc steorate in the composite, and contact angle measurements showed that the hydrophobicity reached a high level of up to 151°, which contributes to the effective selection of oils. The maximum adsorption capacity for cyclohexane was about 22 g/g, indicating a high ability of the material to absorb oil. As a result of the experiments, it was confirmed that ZS@BIF is capable of removing up to 99.9% of oil contaminants from water, and its stability and efficiency were maintained after 10 cycles of repeated use—about 95% of the original performance. It was also found that the material has good magnetic properties, which facilitates its collection after use using a magnetic field. These results demonstrate the high efficiency, repeatability, and practical suitability of the developed sorbent for water purification from oil pollution, which makes it a promising solution for environmental safety and oil spill response.

In the following study [70], superhydrophobic cobalt ferrite (CoFe_2_O_4_) magnetic particles synthesized by the coprecipitation method followed by modification with lauric acid were developed. Structural analysis (XRD, SEM, XPS) confirmed the formation of CoFe_2_O_4_ spinel with micro-nanoscale hierarchical structure. The particles exhibited strong magnetic properties (Ms = 65.52 emu/g) and stable superhydrophobicity (contact angle 157.3°), which was maintained in the pH range of 1–13 and after multiple cycles of use. The functionalized cobalt ferrites were successfully applied for effective separation of water–oil mixtures. In experiments with four organic liquids (hexane, petroleum ether, xylene, olive oil), high efficiency (>94.2%) was achieved, while for a hexane/water mixture the efficiency was 99.6%. The particles showed resistance to repeated use: after 10 separation cycles, the efficiency remained above 93%, and the superhydrophobic properties remained virtually unchanged.

In the paper [71], a new technique for creating superhydrophobic copper meshes for efficient separation of water–oil mixtures was developed. A commercial copper mesh with a surface coating of RTV-1 silicone acting as a binder was used as a base. Carbon nanoparticles (soot) obtained by burning a paraffin candle were uniformly deposited on the silicone film. To increase the hydrophobicity and durability of the coating, the mesh surface was additionally treated with a femtosecond laser, resulting in the formation of micropatterns with hierarchical micro-nanoscale structures. Wetting angle measurements showed that the modified mesh had excellent superhydrophobicity with a water contact angle of 168.9° and a low roll-off angle (5.9°). The resulting superhydrophobic meshes were successfully applied to separate an n-hexane/water mixture in a gravity system. The separation efficiency reached 98% and remained stable for 10 cycles of use. The material also demonstrated high mechanical resistance: after 50 cycles of exposure to a water jet and 5 cycles of a peel test, the surface properties remained virtually unchanged.

### 4.2. Sponges and Foams

In this paper, new approaches to the development of superhydrophobic cellulose papers (SPs) as another class of promising materials for similar tasks are considered. In the review article, various strategies for obtaining SPs were analyzed, including modification of cellulose fibers by deposition of nanomaterials (TiO_2_, SiO_2_, ZnO, Cu(OH)_2_, Fe_3_O_4_) and subsequent treatment with low-energy compounds such as fluoroalkylsilanes or stearic acid. This allowed the formation of a hierarchical micro-nanoscale structure and a significant decrease in the surface energy of the material. Various preparation methods were used: vacuum filtration, spray deposition, self-assembly, phase separation, deep coating, and ATRP. The superhydrophobic papers created demonstrated excellent performance characteristics: the water contact angle exceeded 150°, providing high water-repellent capacity, as well as superoleophilicity, which allowed oil to easily penetrate the structure of the material. Such SPs were used to separate both free water–oil mixtures and complex emulsions. The separation mechanism was based on water retention due to superhydrophobicity (Cassie–Baxter regime) and oil passage according to the Wenzel principle. Some designs made it possible to achieve separation efficiency above 99%, and the flux permeability exceeded 10,000 L m^−2^ h^−1^. The materials demonstrated resistance to acids, alkalis, organic solvents, and mechanical damage and demonstrated the ability to restore superhydrophobicity after damage. In some cases, SPs retained their properties even after 100 separation cycles [72].

In the paper [73], a stable superhydrophobic/superoleophilic melamine foam modified with celery-derived porous carbon (PC) and multi-walled carbon nanotubes (MWCNTs) was successfully developed. The porous carbon was prepared by a green biomass self-activation method, and the nanotubes were synthesized by chemical vapor deposition. The melamine foam was modified by dipping PC or MWCNT with polydimethylsiloxane (PDMS) suspension, followed by heat treatment to fix the components on the surface. The obtained materials possessed a three-dimensional porous structure while maintaining the original pore sizes of melamine foam (150–300 μm). The water contact angles were 159.34° for PC/MF and 156.42° for MWCNT/MF, confirming the pronounced superhydrophobic property. The foams demonstrated high sorption capacity for various oils and organic solvents (chloroform, dichloromethane, ethyl acetate, DMF, hexane, acetone, silicone oil, toluene, and olive, corn and sesame oils), reaching 54–143 g/g for PC/MF and 46–137 g/g for MWCNT/MF. The materials were effectively used for water–oil separation under static and dynamic conditions. The oil absorption process took 3–5 min, after which the aqueous phase remained clean. The foams retained their efficiency after ten cycles of compression and repeated use, demonstrating minimal decrease in sorption capacity. High chemical resistance was also confirmed: superhydrophobic properties and oil absorption capacity did not change after exposure to acidic (pH = 2), alkaline (pH = 12), and salt solutions (3.5% NaCl). Strength tests showed that the materials withstood pressures of up to 31.38 kPa without signs of deformation.

In this work [74], a smart composite membrane based on multi-walled carbon nanotubes (MWCNTs) and titanium nanotubes (TNTs) with superhydrophobic and superoleophilic properties was developed for efficient separation of water–oil mixtures. In the first step, functionalized MWCNTs were coated with titanium dioxide (TiO_2_), followed by hydrothermal conversion into titanium nanotubes. Nitrogen doping provided improved optical properties and affected the morphology and functionality of the composite. The final material was deposited on a nitrocellulose filter substrate using vacuum filtration, forming a thin and durable membrane. The resulting membranes were tested for the separation of various organic pollutants (n-hexane, motor oil, vacuum oil, petroleum ether, toluene) from aqueous mixtures. The membranes demonstrated high superhydrophobicity and superoleophilicity, which ensured efficient passage of the oil phase while completely repelling water. The separation efficiency for all types of oils exceeded 95%, with the CNT-TNT1.0 sample demonstrating the best performance characteristics. The membranes also demonstrated stable efficiency when working with oils of different viscosities, which is an important indicator for real operating conditions. Morphological analysis (SEM, HR-TEM) confirmed the uniform coating of CNTs with titanium nanotubes, and optical studies revealed a narrowing of the band gap due to the interaction of the composite components. Moving on from the previously described three-dimensional superhydrophobic sponges and foams, which have proven highly effective in absorbing oils, this work demonstrates the advantages of creating thin nanocomposite membranes. Such structures provide not only selective water–oil separation, but also resistance to mechanical stress, high chemical stability, and the ability to precisely control surface properties. These features make the developed membranes promising for implementation in industrial wastewater treatment systems and the elimination of oil pollution.

The analyzed research cycle presents modern approaches to the creation of superhydrophobic and superoleophilic materials for the efficient separation of water–oil mixtures, which take into account both the simplicity and cost-effectiveness of synthesis and high operational characteristics in real conditions.

In the early stages, magnetic nanoparticles (CoFe_2_O_4_) and metal meshes modified with carbon nanoparticles and hydrophobic agents were proposed. These materials provided high separation efficiency (>94–99.6%) and ease of regeneration due to magnetic properties or high mechanical strength. Then, superhydrophobic cellulose papers and hybrid sponges on a polyurethane base with multi-walled carbon nanotubes (MWCNTs) were considered. They showed high sorption capacity (up to 86 g oil/g material), resistance to aggressive environments, and stability during repeated use.

Particular attention is paid to the development of environmentally friendly materials based on biomass. Melamine foams modified with porous celery carbon and MWCNTs demonstrated not only excellent hydrophobic–oleophilic properties and high sorption capacity (up to 143 g oil/g), but also resistance to acids, alkalis, and salts. These materials were successfully used for the separation of both light oils and heavy hydrocarbons.

### 4.3. Membranes

Building on this concept, the review article [75] provides a comprehensive assessment of the progress in membrane technologies for water–oil separation, focusing on a wide range of materials and design solutions. The work analyzes various types of membranes: organic (polymer), inorganic (ceramic, metallic), hybrid, metal–organic frameworks (MOFs), nanocomposite, and natural materials. The authors consider key membrane properties, including wettability, permeability, fouling resistance, mechanical strength, and chemical resistance. The developed membranes were used to separate both free water–oil mixtures and complex emulsions of varying viscosities and compositions. Both gravity and pressure filtration methods were used. In some examples, separation efficiencies exceeded 99.9%, and permeabilities ranged from 2000 to 38,000 L m^−2^ h^−1^, depending on membrane structure and operating conditions. Particular attention was paid to developing membranes with high resistance to fouling and the possibility of repeated use: in many cases, efficiency was maintained for 10–100 cycles.

Despite the progress in membrane technologies, the problem of pore clogging and the difficulty of large-scale production remain unresolved. This has led to a search for alternative three-dimensional porous materials with high absorption capacity and mechanical strength. In this context, the work by Liu et al. (2021) proposed the development of a superhydrophobic polyurethane (PU) sponge modified with multi-walled carbon nanotubes (MWCNTs) [76]. A simple one-step technique was used to obtain the material. The surface of pre-treated PU sponges was functionalized using three different silicone agents. Octadecyltrichlorosilane (OTS) was selected as the optimal one, providing the formation of a superhydrophobic surface with a wetting angle of 151.3°. The modification was carried out by ultrasonic dispersion of MWCNTs in toluene with the addition of OTS and subsequent impregnation of the sponges for 24 h. The treatment made the sponges highly water-repellent and also improved their mechanical and thermal properties. The resulting superhydrophobic sponges were used to selectively absorb oils and organic solvents of various viscosities and densities from water. In model tests with six types of oils (soybean oil, kerosene, petroleum ether, chloroform, crude oil, and hexadecane), the material demonstrated an absorption capacity of 14.99 to 86.53 g of oil per 1 g of sponge. The sponge successfully absorbed both light and heavy oils without absorbing water, even with changes in temperature and the ionic strength of the medium. The separation process was carried out both on the water’s surface and underwater: the sponge quickly and completely absorbed the oil, restoring a clean water surface. Mechanical tests showed that the inclusion of MWCNTs made the sponge highly durable and capable of withstanding multiple use cycles. After 10 cycles of absorption and mechanical pressing, the absorption efficiency decreased by less than 10%.

The final stage of the research was devoted to the creation of nanocomposite membranes based on MWCNTs and titanium nanotubes (TNTs). The membranes combined the advantages of previous three-dimensional sorbents with the ability to precisely control the structure, high mechanical strength, and separation efficiency (>95%) for oils of different viscosities under filtration flow conditions.

To facilitate the understanding and comparison of the discussed purification systems, their key characteristics are summarized in Table 3. The table provides a comparative analysis of the superhydrophobic materials and systems described in this section, highlighting their synthesis methods, wettability, oil absorption or separation efficiency, reusability, advantages, and limitations. This summary allows for a clearer evaluation of the strengths and weaknesses of each approach and demonstrates the diversity of strategies developed for efficient oil–water separation.

Thus, the consistent development of technologies—from sorbents with high absorption capacity to membrane structures—demonstrates the evolution of functional materials optimized for various water–oil separation scenarios. The leading areas remain increasing mechanical and chemical resistance, the possibility of repeated use, the environmental friendliness of the original components, and adaptation to industrial operating conditions.

## 5. Prospects and Limitations

Superhydrophobic magnetic nanomaterials (SHMNMs) represent one of the most promising developments in the field of cleaning hydrocarbon contaminants from aqueous media. The unique combination of the properties of these materials—superhydrophobicity, oleophilicity, and magnetic controllability—ensures highly efficient, selective, and reproducible removal of oil and organic contaminants from water [77,78]. However, despite its obvious advantages, this technology also has certain limitations that must be addressed in its further development and implementation.

The key reason for using SHMNMs is their high efficiency of phase separation. Due to the superhydrophobic surface, the materials repel water and simultaneously adsorb oil and organic compounds. This allows achievement of almost complete separation of oil products, even in complex emulsions and wastewater. The surface exhibits minimal water wetting and high selectivity for hydrocarbons, ensuring effective separation [51].

The magnetic properties of these materials open up opportunities for efficient control of their position in the environment. The use of an external magnetic field makes it easy to collect used sorbents, move them to the spill area, or remove them from the system after the cleaning process is complete. This simplifies operation and reduces maintenance costs compared to traditional mechanical or gravity separation methods [6].

In addition, the technology is highly reusable. Modern materials withstand numerous sorption and desorption cycles without significant deterioration of their hydrophobic and magnetic characteristics. This makes them economically attractive for industrial use [47].

Of great importance is the ability to adapt superhydrophobic magnetic nanomaterials to various operating conditions. Compositions and coatings have been developed that can retain their functional properties over a wide pH range, in the presence of aggressive chemicals, and when exposed to temperature fluctuations [48]. Such stability expands the scope of application of the technology from emergency oil recovery to continuous operation in treatment facilities and industrial filtration systems.

Another promising area is the development of materials based on renewable raw materials. The use of biomass and green synthetic methods reduces the carbon footprint and the potential impact on the environment. This meets modern requirements for sustainable development and environmental safety [78]. To address the high cost and environmental concerns of fluorine-based hydrophobic coatings, recent studies have proposed the use of natural waxes and biopolymers (such as chitosan and polylactic acid) as eco-friendly and biodegradable alternatives, while maintaining adequate superhydrophobic properties [16]. Similarly, biodegradable matrices and protective coatings have been proposed to reduce the risk of nanoparticle leaching and bioaccumulation in aquatic environments [79]. These strategies contribute to minimizing the ecological footprint of superhydrophobic materials while maintaining their functional performance.

Despite many advantages, there are certain limitations that must be taken into account when developing and using superhydrophobic magnetic nanomaterials.

One of the main problems is the complexity and high cost of production of certain types of materials. Some synthesis methods require specialized equipment, precise control of conditions, and the use of expensive chemical reagents. This can significantly increase the cost of the final products, especially when using complex multilayer or hierarchical structures. Mechanical stability remains an important factor. Superhydrophobic coatings may be subject to abrasion, cracking, or degradation with repeated use or mechanical stress. Materials that operate under conditions of mixing, filtration, or contact with abrasive particles are particularly vulnerable.

A significant limitation is the durability of superhydrophobic properties. Hydrophobic modifiers, especially organic compounds, can gradually lose activity under the influence of ultraviolet radiation, oxidative processes, or aggressive chemicals present in wastewater or seawater. This requires regular restoration or renewal of functional coatings.

Separately, it is necessary to note the potential environmental risks associated with the possible release of nanoparticles into the environment. Despite the stability of many modern materials, their wear during operation can lead to the migration of nanoparticles beyond the working area. This creates risks of bioaccumulation and toxic effects on aquatic organisms, especially if non-degradable synthetic components are used.

It is also worth considering the limitations of operating conditions. Some materials show reduced performance under extreme conditions, such as very high or low pH, prolonged exposure to high temperatures, or the presence of strong oxidizing agents. This limits the use of some types of nanomaterials in specific industrial environments.

Finally, there is a need to standardize methods for evaluating the performance and safety of superhydrophobic magnetic nanomaterials. The diversity of approaches to synthesis and testing makes it difficult to compare results from different research and development efforts, slowing down the commercialization of the technology [51].

Thus, superhydrophobic magnetic nanomaterials have significant potential to increase the efficiency and sustainability of oil and water separation processes. They combine unique functional properties that allow solutions to the problems of cleaning aqueous media with high accuracy and minimal costs. At the same time, the successful introduction of such materials into industrial practice requires further improvement of synthesis methods, increasing the mechanical strength and durability of coatings, and the development of environmentally friendly and economically viable solutions. Further research should aim to overcome existing limitations and develop new generations of materials with improved characteristics and minimal impact on the environment. An integrated approach, including materials science, nanotechnology, environmental engineering, and economic analysis, will be the key to the successful development of this technology in the coming years.

Despite the promising properties of the reviewed methods, several challenges limit their industrial deployment. The sol–gel process, while producing stable and chemically resistant coatings, is brittle and incompatible with flexible or soft substrates, which precludes its use on textiles or foams. Electrospinning offers scalable production of porous membranes, but the need for precise control and post-treatment complicates large-scale implementation. Dip-coating is simple and scalable but prone to non-uniformity and defects if not carefully optimized. Laser-based methods provide precise surface structuring but require expensive and specialized equipment, making them economically unfeasible for large-scale operations. Furthermore, the widespread use of fluorine-containing hydrophobic agents raises environmental and regulatory concerns due to their persistence and toxicity. These factors explain why many of the laboratory-scale solutions have not yet been translated into cost-effective, industrially viable technologies.

Recent toxicological studies have demonstrated measurable adverse effects of magnetic nanoparticles on various aquatic and terrestrial organisms, emphasizing the importance of assessing long-term ecological consequences. For instance, Keshta et al. (2024) report that iron oxide nanoparticles can cause oxidative stress and membrane damage in algae and fish at concentrations above 10 mg/L [17]. Rezaei and Hassanajili (2023) also found that bio-waste-based magnetic nanomaterials, while effective in oil spill cleanup, release fine particles that accumulate in sediments and reduce microbial diversity in soils [24]. Long-term persistence of fluorinated superhydrophobic coatings and non-biodegradable components raises concerns about soil fertility loss and food chain disruption [79]. These findings underscore the need to design SHMNMs using biodegradable matrices and conduct thorough risk assessments before large-scale deployment.

To overcome these challenges and unlock the full potential of SHMNMs, it is essential to identify and pursue specific research directions that can improve their functionality, environmental safety, and industrial applicability. Future research should focus on several promising avenues. One important area is the development of bio-inspired hierarchical structures that mimic natural superhydrophobic surfaces (e.g., lotus leaf, springtail skin) to enhance durability and self-healing capabilities. Novel synthesis methods such as 3D printing and layer-by-layer assembly could provide better control over surface morphology and scalability. The design of fluorine-free and biodegradable superhydrophobic coatings remains a key challenge to reduce environmental impact, and research into natural waxes, biopolymers, and hybrid organic–inorganic systems is encouraged. Furthermore, the integration of SHMNMs into membrane bioreactors and advanced filtration systems could enhance the efficiency of wastewater treatment beyond oil–water separation. Emerging application areas such as anti-icing surfaces for transportation infrastructure and biomedical devices (e.g., antibacterial coatings, controlled drug release) also deserve investigation. These directions offer exciting opportunities for expanding the utility of SHMNMs while addressing current limitations.

To address scalability and cost issues, recent studies have proposed roll-to-roll coating, spray-coating, and dip-coating techniques as scalable alternatives to laboratory-scale methods like sol–gel and laser processing, enabling continuous fabrication on large areas (e.g., [80,81]). The use of natural waxes, biopolymers, and inexpensive silica-based materials in place of fluorinated compounds significantly reduces production costs and improves environmental compatibility [24]. Pilot-scale demonstrations of superhydrophobic magnetic materials for oil spill cleanup in coastal waters and industrial wastewater treatment have confirmed their practical applicability while highlighting areas for further optimization [82,83]. These strategies demonstrate viable pathways for overcoming current limitations and bringing SHMNMs closer to industrial implementation.

At the same time, ensuring the environmental safety and regulatory compliance of SHMNMs is crucial for their widespread adoption. International standards and guidelines, such as those developed by the U.S. Environmental Protection Agency (EPA) and the European Chemicals Agency (ECHA), provide a framework for assessing the potential risks associated with nanoparticle release and long-term environmental impact. Recent advances also emphasize the use of green synthesis strategies, including biodegradable matrices, natural modifiers, and environmentally friendly solvents. For example, Keirouz et al. demonstrated the sustainable fabrication of graphene-based nanofibrous membranes using the green solvent Cyrene, showcasing how high performance can be combined with environmental responsibility [84]. Incorporating such strategies and adhering to regulatory standards are essential steps toward the safe and practical application of SHMNMs.

Together, these technological advances, regulatory considerations, and sustainable synthesis approaches form a comprehensive roadmap for translating SHMNMs from laboratory concepts to viable industrial solutions. Continued interdisciplinary research integrating materials science, engineering, environmental assessment, and economic analysis will be key to overcoming remaining challenges and unlocking the full potential of these materials for environmental and industrial applications.

## 6. Conclusions

The development and application of superhydrophobic magnetic nanomaterials (SHMNMs) for oil and water separation is a dynamically developing area at the intersection of environmental and materials science technologies. These materials successfully combine superhydrophobicity, which enables effective water repellency; oleophilicity, which ensures selective absorption of hydrocarbons; and magnetic properties, which facilitate the collection and reuse of sorbents. The synthesis methods discussed in the article —including the sol–gel process, electrospinning, the immersion (dip-coating) method, laser–chemical processing, and the use of biomass—each have distinct advantages and limitations that determine their applicability.

Specifically, the sol–gel method provides stable, corrosion-resistant coatings with controlled composition and high chemical resistance, but is unsuitable for flexible or soft substrates due to the brittle nature of the resulting films. Electrospinning allows the fabrication of flexible, porous membranes with high surface area and good scalability, although it requires post-treatment and precise control of process parameters. The immersion method is simple, cost-effective, and easily scalable, making it suitable for coating sponges, fabrics, and meshes, yet it can lead to defects if solution properties and immersion speed are not optimized. Laser–chemical processing enables precise, localized modification of surfaces on metals, glass, or polymers without the need for templates, but is limited by the high cost and specialized equipment required. The use of biomass offers an environmentally friendly and low-cost alternative, producing materials with high sorption capacity and good magnetic controllability, although mechanical strength may be limited depending on the chosen biomass source.

Modern studies confirm the high efficiency of SHMNMs in removing oil products and organic pollutants from water, with materials demonstrating stability in aggressive environments, reusability over multiple cycles, and adaptability to various industrial and natural systems. The incorporation of environmentally friendly raw materials, such as biomass and biogenic nanoparticles, enhances the sustainability of the technology and mitigates environmental risks.

However, several challenges remain to be addressed. It is necessary to improve the mechanical robustness of coatings to prevent wear and damage under operational conditions, to enhance the durability of superhydrophobic properties under UV and chemical exposure, to optimize production processes for reduced costs and better scalability, and to standardize criteria for evaluating material effectiveness and environmental safety. Special attention should also be paid to assessing and minimizing potential environmental impacts associated with nanoparticle migration during material wear.

In conclusion, SHMNMs show significant promise for deployment in wastewater treatment systems, emergency oil spill response, and industrial filtration. Continued interdisciplinary research—focusing on overcoming current limitations, developing environmentally benign and cost-effective materials, and refining manufacturing techniques—will drive progress in this field and expand the practical application of these advanced technologies.

## Figures and Tables

**Table 1:** Comparative characteristics of bases for superhydrophobic materials.

Base Type	Contact Angle (°)	Stability	Compatibility with Magnetic Nanoparticles	Advantages	Limitations	Sources
Inorganic (SiO_2_, TiO_2_)	>150 (depending on method)	Medium, requires protection from abrasion	Low, additional modification of Fe_3_O_4_ is required	Ease of creating microstructure, high chemical resistance	Fragility, poor adhesion to soft substrates	[38]
Polymer (PDMS, PU)	160–167	High, withstands up to 30 abrasion cycles	Average, magnetic particle encapsulation is possible	Flexibility, compatibility with various surfaces	Limited thermal stability, requires modification	[39]
Carbon materials (rGO, CNT)	162–165, stable in pH tests	Moderate, depends on the modification method	Good, rGO and CNTs are easily modified with Fe_3_O_4_	High specific surface area, chemical stability	Additional functionalization is required	[40,41]
Hybrid structures (rGO + Fe_3_O_4_, polymer + oxide)	Up to 162, stable after 10 cycles	High, retains properties after repeated use	Excellent, structure is designed to incorporate Fe_3_O_4_	Combination of strength and functionality	Complexity of synthesis and distribution of components	[42,43]

**Table 2:** Modern methods of synthesis of superhydrophobic materials.

Method	Principle	Advantages	Disadvantages	Examples of Achieved Properties	Sources
Sol–gel method	Hydrolysis and polycondensation of precursors (TEOS, GPTMS, etc.) → formation of oxide network → application to substrate → modification with organofluorine	Ease of obtaining nanostructures; controlled composition; chemical resistance	Fragility of the resulting coatings; not always compatible with soft substrates	Contact angle > 160°; corrosion and chemical resistance; applicable with Fe_3_O_4_, TiO_2_	[45,46]
Electrospinning	Pulling a polymer solution in an electric field → formation of nanofibers → modification with a hydrophobic agent or introduction of nanoparticles	Creation of porous membranes; high surface area; substrate flexibility	Post-processing is required; difficulty in uniformly coating large areas	Contact angle 160–167°; stability after 10+ cycles; compatible with Fe_3_O_4_, rGO	[50,51]
Dip-coating method	Substrate immersion in solution → nanoparticle deposition → drying/curing	Ease of implementation; suitable for sponges, fabrics, meshes; scalability	Dependent on viscosity, speed, concentration; possible defects	Wetting angles 158–162°; stability in pH 3–11; high separation efficiency (up to 99.8%)	[56]
Laser–chemical treatment	Localized ablation with femtosecond laser → formation of microstructure → chemical modification with stearate	Precision; versatility (works with metal, glass, polymer); does not require templates	Requires laser equipment; high cost; not always compatible with magnetic components	Contact angle > 160°; maintained after heating and washing cycles	[61]
Use of biomass	Preparation of biomatrix (carbonization/heat treatment) → introduction of Fe_3_O_4_ → hydrophobization (PDMS, stearate)	Environmentally friendly; low cost; magnetic controllability; reusability	Limited mechanical strength; requires selection of biomaterial	Contact angle > 150°; sorption 45–70 g/g; magnetic sensitivity; stability > 10 cycles	[64]

**Table 3:** Comparative analysis of superhydrophobic materials for oil–water separation.

Material/System	Synthesis Method and Components	Wettability (Contact Angle)	Oil Absorption/Separation Efficiency	Stability	Reusability (Cycles)	Advantages	Limitations
ZS@BIF sorbent [69]	Zinc stearate on BIF via coating	151°	22 g/g (cyclohexane), 99.9% removal	Stable up to 60 days	~10 cycles (~95% retained)	High efficiency, magnetic recovery	Limited to light oils
CoFe_2_O_4_ particles [70]	Co-precipitation + lauric acid	157.3°	94–99.6% for various oils	Moderate	>10 cycles (>93% retained)	Wide pH stability, magnetic	Need for post-modification
Cu mesh [71]	RTV-1 + soot + femtosecond laser	168.9°	98% separation	High chemical resistance	>10 cycles	High hydrophobicity, durable	Requires laser equipment
Cellulose papers [72]	Cellulose + nanoparticles + silanes	>150°	>99% separation, >10,000 L/m^2^·h flux	Moderate	Up to 100 cycles	High permeability, chemical resistance	Fragility
PU sponge + MWCNT [73]	Silicone + MWCNTs	151.3°	14.99–86.53 g/g	Good under washing/heating	>10 cycles (<10% loss)	Absorbs light and heavy oils, durable	Lower precision of structure
Melamine foam + PC/MWCNT [74]	Biomass carbon or MWCNT + PDMS	156–159°	46–143 g/g	High chemical resistance	>10 cycles	High sorption, resistant to pH, salt	Biomass source needed
CNT–TNT membrane [75,76]	MWCNT + TiO_2_ nanotubes + doping	>155°	>95% separation	Flexible, good stability	Stable in flow systems	High mechanical strength, thin films	Complex fabrication

## Data Availability

No new data were created or analyzed in this study.
